# A general-purpose signal processing algorithm for biological profiles using only first-order derivative information

**DOI:** 10.1186/s12859-019-3188-4

**Published:** 2019-11-27

**Authors:** Yuanjie Liu, Jianhan Lin

**Affiliations:** 1 College of Information and Electrical Engineering, China Agricultural University, Haidian, Beijing, 100083 People’s Republic of China; 2 Key Laboratory of Agricultural Information Acquisition Technology, Ministry of Agriculture and Rural Affairs, China Agricultural University, Haidian, Beijing, 100083 People’s Republic of China

**Keywords:** Passing accumulation, First derivative, Baseline correction, Signal extraction

## Abstract

**Background:**

Automatic signal-feature extraction algorithms are crucial for profile processing in bioinformatics. Both baseline drift and noise seriously affect the position and peak area of signals. An efficient algorithm named the derivative passing accumulation (DPA) method for simultaneous baseline correction and signal extraction is presented in this article. It is an efficient method using only the first-order derivatives which are obtained through taking the simple differences.

**Results:**

We developed a new signal feature extracting procedure. The vector representing the discrete first-order derivative was divided into negative and positive parts and then accumulated to build a signal descriptor. The signals and background fluctuations are easily separated according to this descriptor via thresholding. In addition, the signal peaks are simultaneously located by checking the corresponding intervals in the descriptor. Therefore, the eternal issues of parsing the 1-dimensional output of detectors in biological instruments are solved together. Thereby, the baseline is corrected, and the signal peaks are extracted.

**Conclusions:**

We have introduced a new method for signal peak picking, where baseline computation and peak identification are performed jointly. The testing results of both authentic and artificially synthesized data illustrate that the new method is powerful, and it could be a better choice for practical processing.

## Background

In profile-based bioinformatics data analysis, digitized signals have aroused universal interest with a broad range of applications. Extraction of qualitative and quantitative information in a large number of analytical signals from the background noise, however, poses significant challenges. In order to obtain accurate and clear results, some effective methods should be proposed and implemented to perform signal extraction before further data analysis. For instance, mass spectrometry is one of the most used tools to analyze large biological molecules, where the meaningful conclusion of the proteomic studies depends on the extracted signal peaks. In health care, chemical sensing relies on various spectroscopic techniques, which are not meaningful until the signals are extracted [1]. In agricultural applications, such as audio sensing for animal monitoring [2], the situation is exactly the same. Examples in bioelectrical activity measurements, such as electrocardiograms (ECG) and electroencephalograms (EEG) [3], mainly depend on wavelet analysis for signal processing [4].

Two parameters of the signal are often studied: peak position and peak area. Sometimes, the shape of signal is further studied through detailed analyses. In general, we can decompose a signal peak detection procedure into three consequent parts: smoothing, baseline correction and peak finding. Baseline removal and signal extraction are the core problems in signal processing. The baseline drift comes from the background fluctuation that appears as slow but large-scale ups and downs. It is a kind of low-frequency noise. When the signal peaks are selected according to their height, width or shape, the distortion and vertical shift caused by the baseline drift result in a significant interference. The high-frequency noises introduce rapid small-scale fluctuations. When peaks are identified as the signals, these noises harm the integrity of signal peaks while generating many interfering peak points. Various methods to smooth the noise and correct the drift with signal reception have been developed. However, due to randomness and complexity, the robust and accurate signal picking remains a challenging task. In this context, we propose a new signal feature extraction algorithm from a raw profile. After comparison with several classical methods, using various kinds of spectra and synthesized profiles, the proposed method was found to be accurate, flexible and easy to use.

### Previous works

Baseline drift and random noise are common degradation problems in signal detection [5, 6]. Several methods based on various theories have been developed to solve these two problems. For pure computational methods, the need to extract key signal features that enable advanced processing algorithms to discover useful contextual information has led to the development of a wide range of algorithmic approaches. These include using Fourier analysis, wavelet analysis, the least squares method, computational geometry, neural networks and so on. Generally, after the removal of baseline drift, the signal peak identification could be focused on extraction using its width or area against the noise interference. Baseline correction thus is used as the main component of signal processing. This is especially true when instruments are being used to detect chemical reactions. Actually, an important series of algorithms has been developed in analytical chemistry in which numerous types of spectra are primitively used. There is a long history of developing numeric algorithms for processing the mass, fluorescent, or infrared spectroscopies. Shirley backgrounds [7], airPLS [8], AIMA [9], and Orthogonal Basis [10] are classic techniques playing important roles in different applications and subjects. The unified variation model [11], LMV-RSA [12] and the techniques based on neural networks [13], singular analysis [14], optimization method [15] and computational geometry [16] can also achieve improved effectiveness or efficiency, and some are able to perform joint baseline-correction and denoising.

In electronic signal processing, wavelet methods are widely applied. For example, in audio processing, multilevel 1-D wavelet analysis [17] is typically performed for denoising. In ECG data processing, the EMD method plays a dominant role [18]. It has made significant contributions to the development of wearable health care systems for breathing, cardiology and temperature measurements.

### Our work

In this article, we proposed a fully automatic scheme using only the first-order derivative. Others attempted to use the derivative for signal processing [19], where both first and second derivatives were utilized. However, the effect was not good enough and the derivative method was not widely accepted.

In our algorithm, we used only the information of the first derivative and built a straightforward procedure that was able to simultaneously remove the baseline drift and extract the peak signals. It was named derivative passing accumulation (DPA) since it was based on the application of accumulation on the first derivative. The proficiency of this new method was mainly driven by the excellent properties of the derivative. Compared to the previous ones, this new algorithm is cleaner, more vigorous and more efficient.

## Results

We selected three representative classical algorithms for comparison with our DPA method, i.e. wavelet [20], EMD [18] and airPLS [8]. These three algorithms were acknowledged as the most commonly used methods in processing electronic and spectroscopic signals.

For the testing data, we chose mass spectroscopy, Raman spectroscopy, audio, ECG and infrared spectroscopies. These were the typical data forms in biological measurements. As had been explained above, in one-dimensional profile processing, the baseline correction is the most important step. For methods based on thresholding separation, the signal peak picking is close to a completion of whole processing after the effective baseline removal. Because we do not know the precise signal information on these authentic data, to better present the comparison, we will only implement the baseline detection procedure in this part of testing. The results intuitively illustrated the performance of the algorithms.

Then, we presented the analysis of the signal location accuracy and peak area loss based on artificially synthesized data to numerically measure the effectiveness.

### Testing on authentic data

**Fig. 1:**
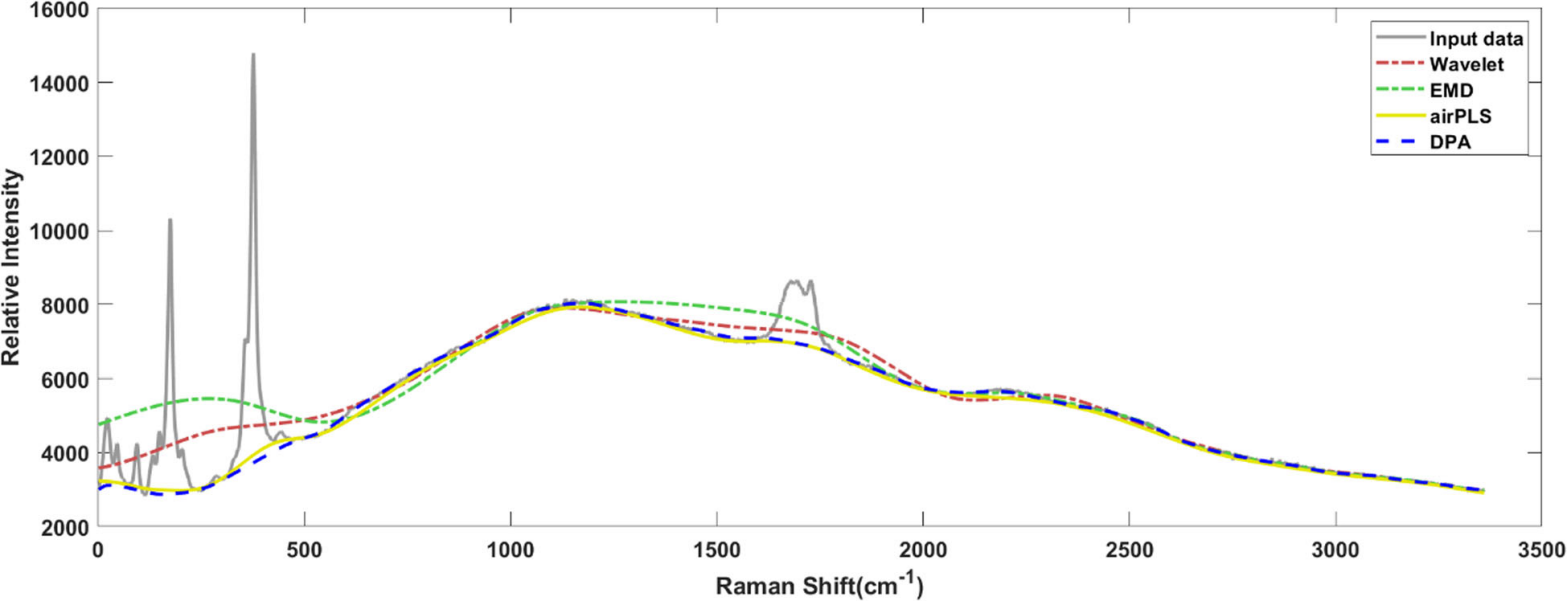
The methods are implemented on the Raman spectroscopy for baseline detection. The DPA method generates a baseline similar to the airPLS method and much better than the EMD and wavelet method

The results of the four algorithms with respect to the Raman spectroscopy curve were shown in Fig. 1. The testing data-trace was a spectrum from RRUFF database [21]. It could be claimed from the results that the DPA method apparently outperformed the EMD and the Wavelet methods. At the same time, the DPA method generated a very close result to the airPLS method, which was the most widely used method specialized for baseline detection in spectroscopy processing.

**Fig. 2:**
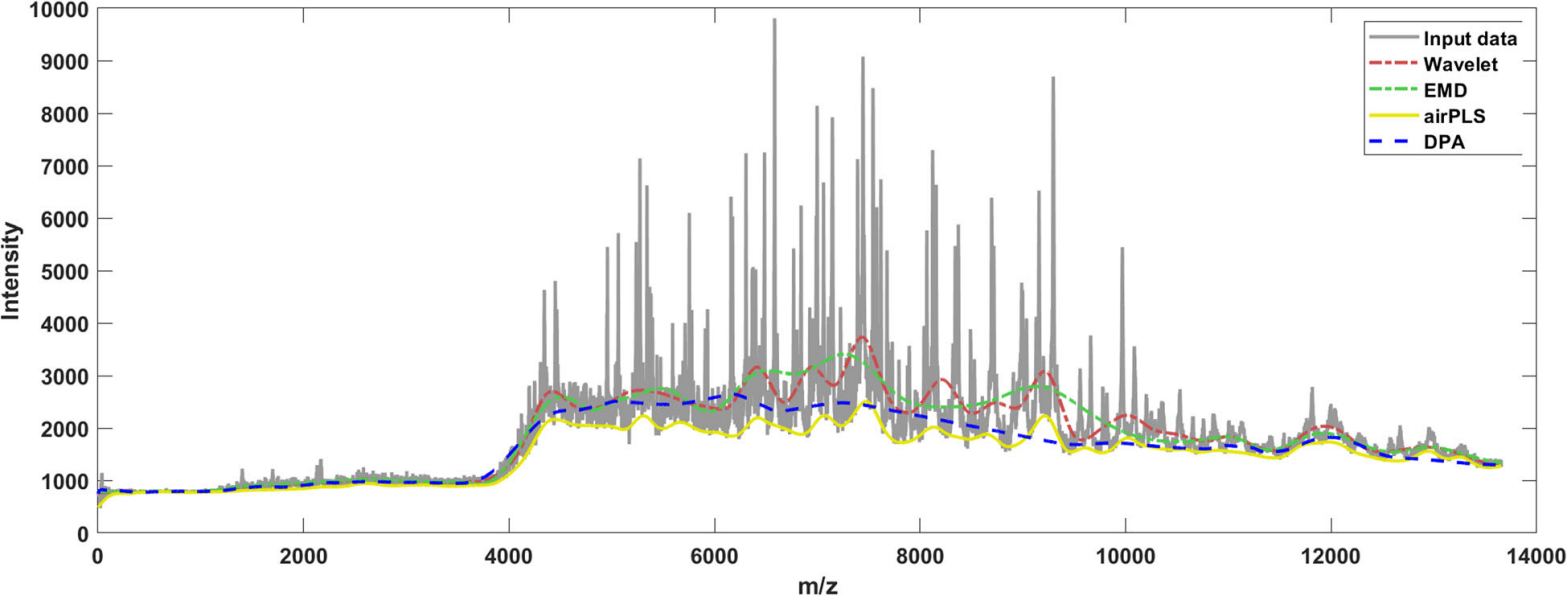
The methods are implemented on the mass spectrum to detect the baseline. The DPA method captured the basic trend of the drift

The results with respect to the mass spectroscopy data are shown in Fig. 2. The testing data-trace is a MALDI-TOF mass spectra produced in bacteria protein analysis. The EMD and the Wavelet methods generated overestimated baselines. The airPLS method generated a dental baseline which was not preferred. The DPA method captured the basic trend of the drift better.

**Fig. 3:**
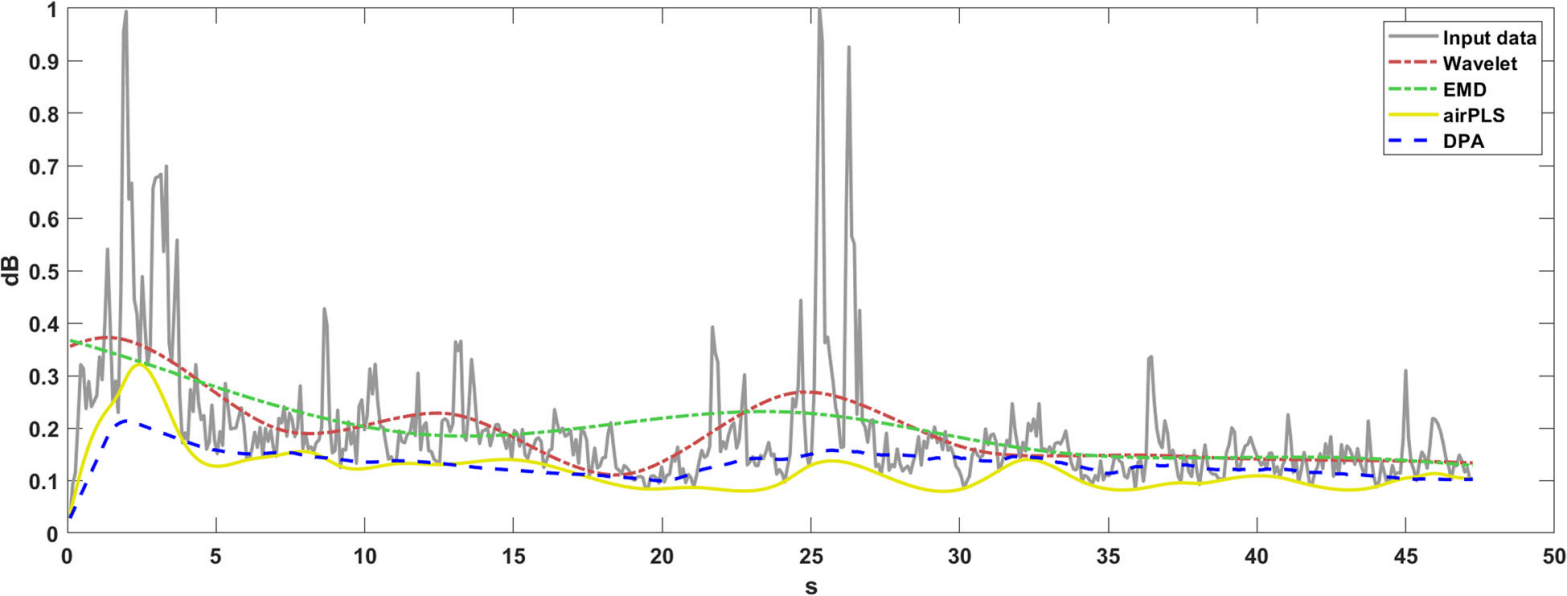
The baselines detected by the comparison algorithms on the energy curve of pig farm audio data

The results of the four algorithms with respect to the energy curve of a piece of audio are shown in Fig. 3. The audio data were taken from the monitoring of a pig farm.

**Fig. 4:**
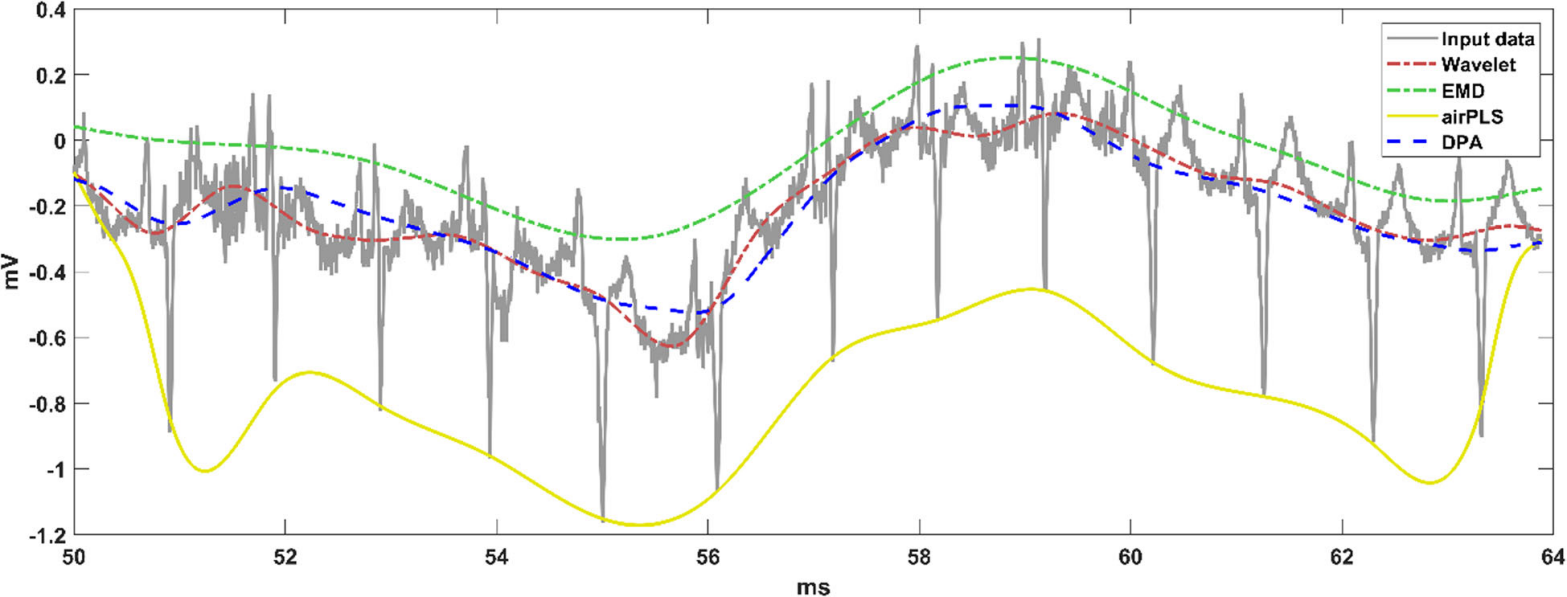
The baseline detections implemented on the ECG data using the comparison algorithms. The DPA method works well and the result of the airPLS method on this data-trace is invalid

**Fig. 5:**
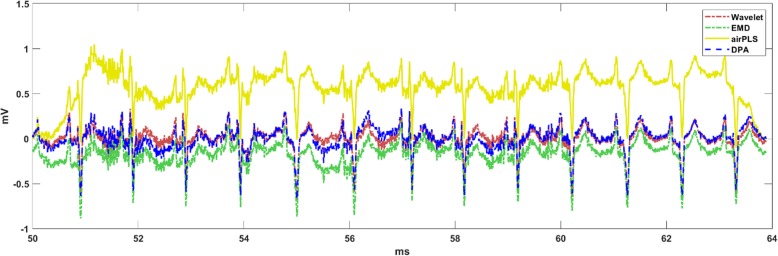
The baseline corrections implemented on the ECG data using the comparison algorithms. The waveform appears more stable after the baseline removal using the DPA method

The results on the ECG data are shown in Fig. 4. The data were downloaded from the MIT-BIH Arrhythmia Database [22, 23]. From Fig. 4, it is apparent that the airPLS performed poorly when processing ECG data. The corresponding baseline corrected results are shown in Fig. 5. It is reasonable to conclude that the waveform was more stable after the baseline removal using the DPA method.

**Fig. 6:**
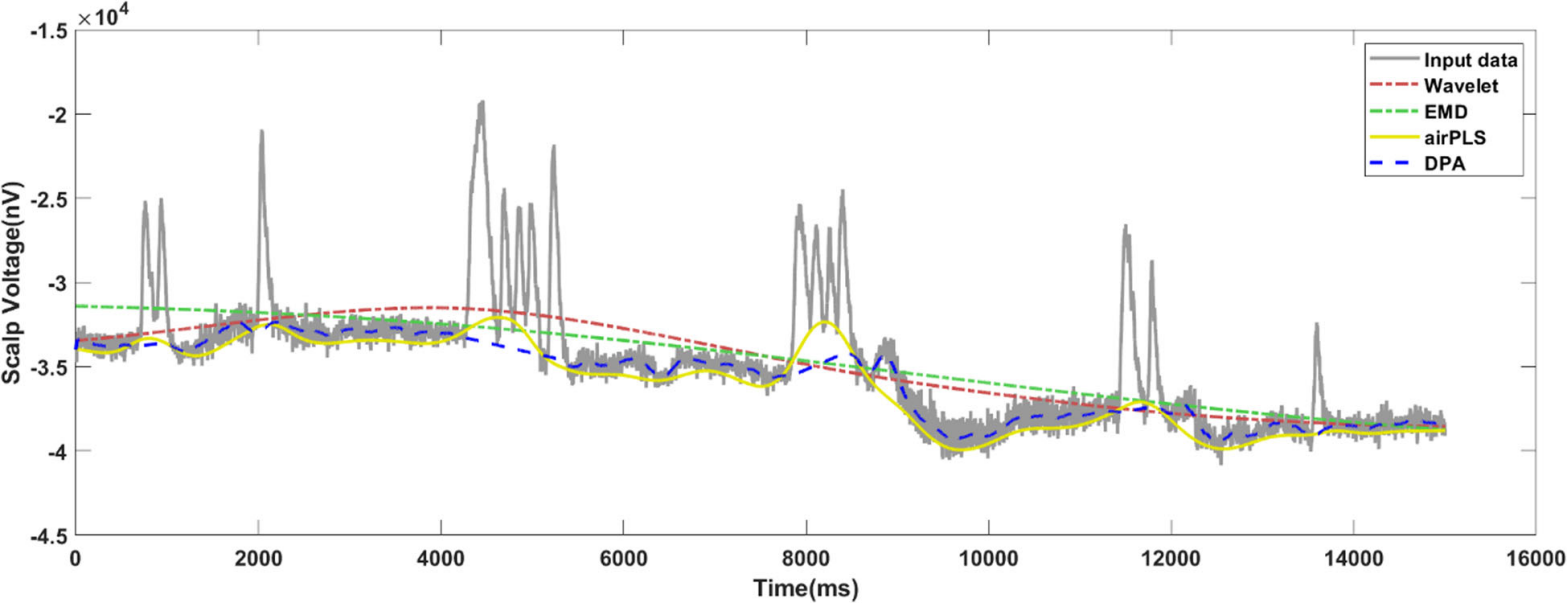
The baseline detection results for the motor imagery EEG data using the comparison algorithms. The DPA method generates a more accurate result

Moreover, on the motor imagery EEG signal, which had been getting more attractive along with the rising of Brain Computer Interface, the DPA method also outperformed its companions. The results tested on part of motor imagery signal of the left-hand movement are presented in Fig. 6. The testing data were taken from the Project BCI - EEG motor activity data set.

**Fig. 7:**
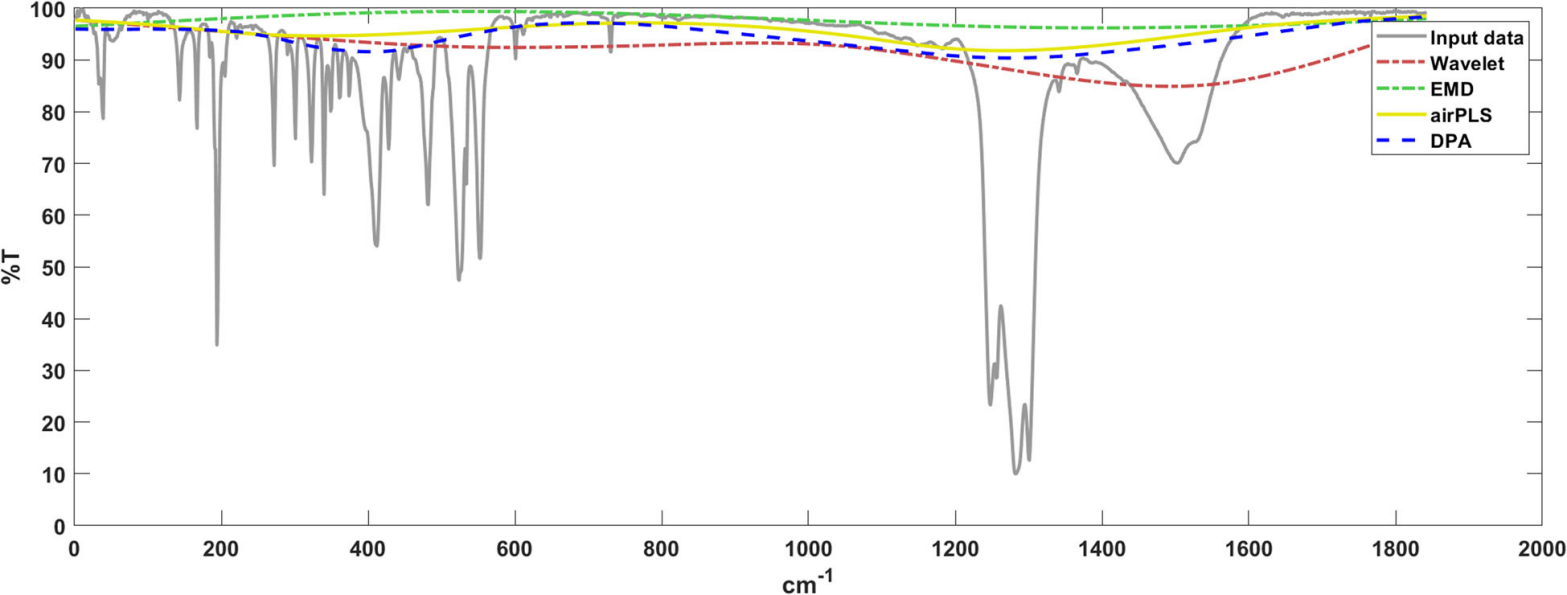
The baseline detection results for the infrared spectrum using the comparison algorithms. The airPLS and DPA methods performed similarly and are better

The results of the infrared spectroscopy are shown in Fig. 7. Infrared spectroscopy is a standard method for detecting organic matters. The organic compound could be qualitatively analyzed through infrared spectroscopy whether it was a gas, liquid or solid. In biochemical measuring, infrared spectroscopy is a basic and necessary technique. The position, number, absorption intensity and shape of the peaks in the infrared spectrum are related to the structure and state of the compound. In addition, the baseline drift is ubiquitous with the infrared spectra, which dramatically affects the peak detection. As such, the processing algorithm is important. From the results, we claim that the airPLS and DPA methods performed similarly and were better than two others, while the wavelet method produced undercut baselines and the EMD method produced overcut baselines.

### Testing on synthesized data

In addition to the authentic data that can be used to illustrate the practical performance of the new method, we carried out detailed analysis on the peak identification via artificially synthesized data in which the information of the simulated signal peaks was precisely known.

The synthesized data were generated by adding three parts together: the long, softly fluctuating waveform simulating the baseline drift; the sharp spikes with different widths and heights to simulate the signal peaks; and the white noise. The curve simulating the baseline was constructed by using the Fourier series. According to the mathematical theorem, any function *f*(*x*) could be represented by the Fourier series expansion.

$$ f(x)={a}_0+\sum \limits_{k=1}^{\infty}\left({a}_k\cos \kern0.2em k{\omega}_0x+{b}_k\mathit{\sin}\kern0.2em k{\omega}_0x\right) $$

**Fig. 8:**
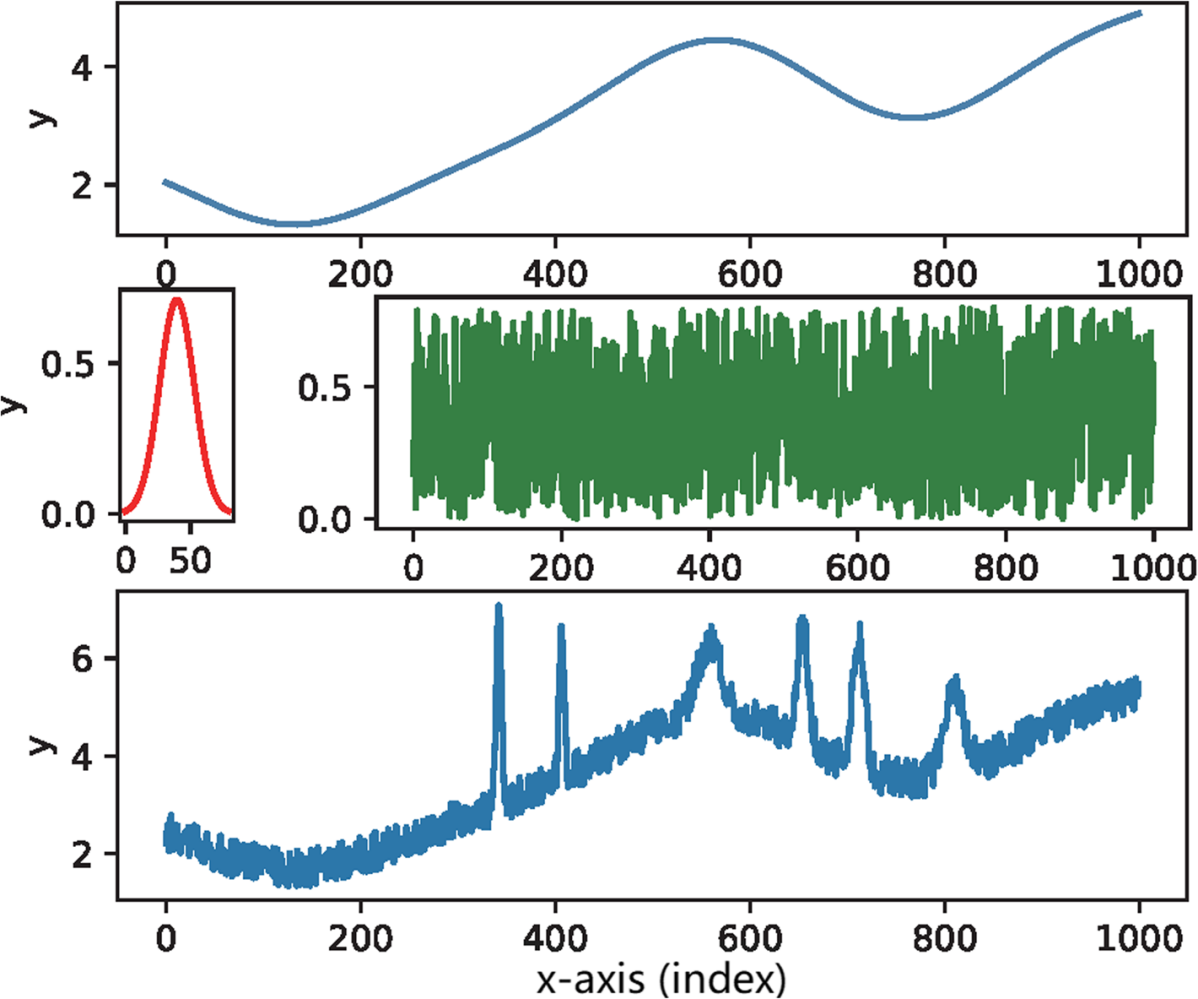
Upper: the baseline, Middle left: the signal peak, Middle right: the noise, and Bottom: the synthesized data-trace

We randomly selected some long periods with random coefficients in the summation. In this way, a slowly undulating waveform was produced. We could theoretically guarantee that the simulating baselines were sampled from a broad scope. The signals were simulated using the Gaussian peak, which was a widely applied standard model. Universal peaks with different heights and widths could be produced by randomly choosing the mean and variance in the Gaussian model. The noise was generated by sampling in a uniform distribution. The typical synthesized data are shown in Fig. 8.

We implemented the four methods to remove the baseline drift. The performance of the methods was measured by the peak area loss rate. Since the position and area of the signal peak were precisely known, the peak area in the corrected trace could be calculated at the preset peak location. The loss rate of each algorithm is given by comparing the results with the preset peak area. Since the classical wavelet, EMD and DPA method were able to locate the signals directly, we examined the peak identification performance in addition.

**Fig. 9:**
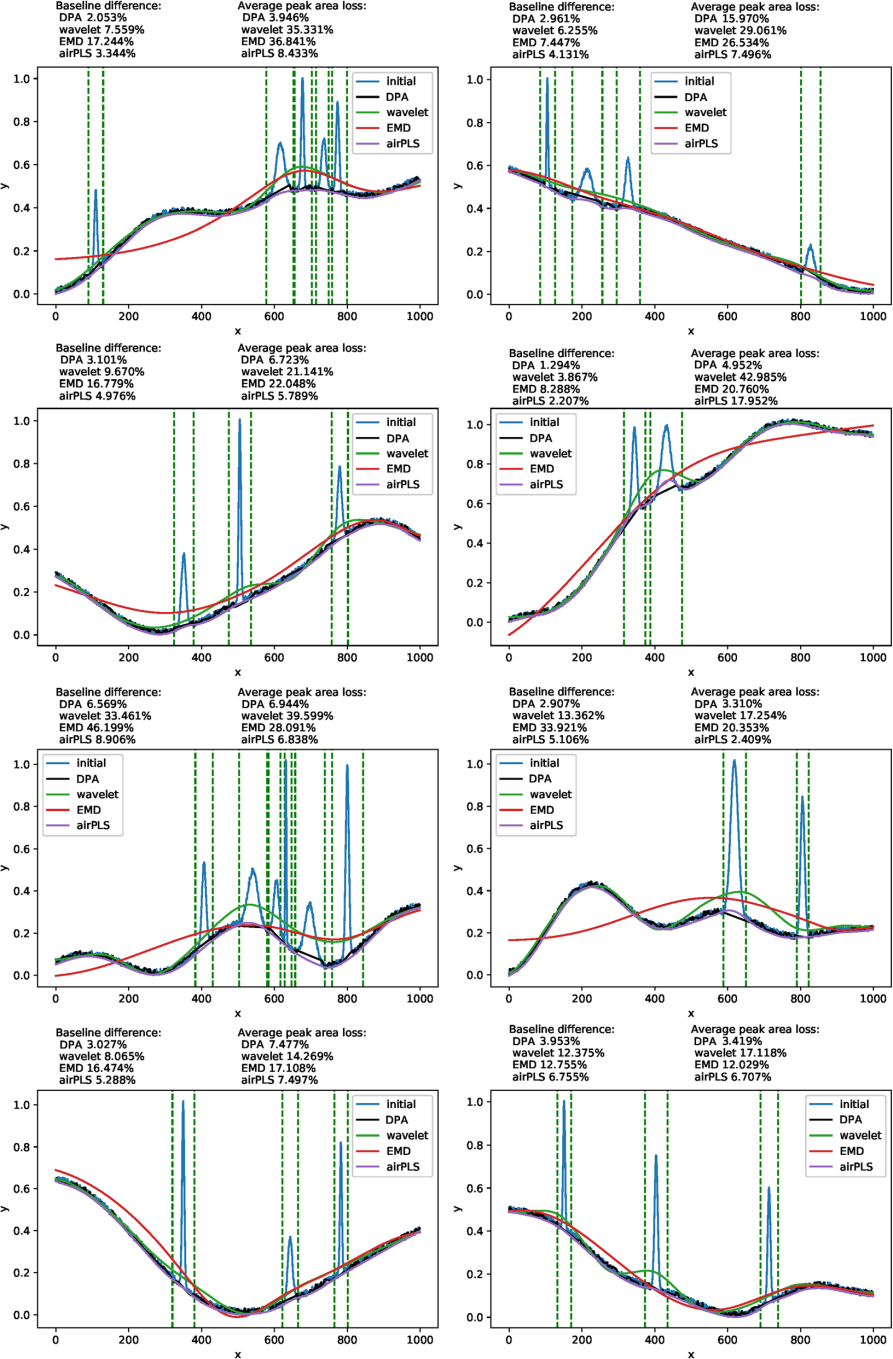
The testing results of the four comparison methods implemented on randomly synthesized simulated signal traces

The results were graphically presented in Fig. 9. From the results, it could be concluded that the DPA method performed better than the other comparative methods in the baseline detection. The error of the baseline that unfortunately occurred in the signals’ interval caused the peak area loss. In this regard, the DPA outperformed the wavelet and EMD methods but not the airPLS. As the famous baseline correcting method, the airPLS method was outstanding in most occasions. Its disadvantages were that it was not able to process the ECG data and the signal peak could only be extracted a step behind rather than simultaneously.

As mentioned above, except for the airPLS method, the classical wavelet, EMD and our DPA methods were implemented based on transforming. The three methods were able to directly locate the signal peaks by setting the threshold in the transformed intermediate representation. We carried out a set of experiments on these three methods to test the detection accuracy (as measured by the peak missing rate) and area loss rate on the correctly recognized peaks. The numerical results are presented in Table 1. From this set of results, we claim that the DPA method performed better than its two companions with respect to the signal extraction precision.

**Table 1:** the peak extraction accuracy. For both peak missing rate and area loss rate, the lower the better

Testing Number	Method					
	DPA		Wavelet		EMD	
	peak missing rate	area loss rate	peak missing rate	area loss rate	peak missing rate	area loss rate
1	0.00%	7.29%	0.00%	15.74%	0.00%	11.82%
2	0.00%	9.86%	0.00%	15.29%	0.00%	12.09%
3	0.00%	13.47%	0.00%	21.04%	0.00%	19.25%
4	0.00%	17.20%	10.71%	13.76%	3.57%	19.18%
5	0.00%	15.68%	0.00%	36.56%	0.00%	9.27%
6	0.00%	19.16%	0.00%	26.74%	0.00%	19.07%
7	3.45%	2.57%	0.00%	19.55%	0.00%	24.07%
8	0.00%	3.13%	0.00%	26.02%	18.18%	17.21%
9	0.00%	8.62%	0.00%	36.43%	0.00%	36.02%
10	0.00%	4.72%	0.00%	35.12%	0.00%	28.44%
11	0.00%	11.26%	0.00%	47.87%	0.00%	16.22%
12	0.00%	3.60%	0.00%	44.72%	0.00%	8.37%
13	0.00%	9.21%	0.00%	39.72%	0.00%	15.70%
14	0.00%	8.78%	3.33%	35.35%	0.00%	10.60%
15	0.00%	6.83%	0.00%	16.24%	0.00%	38.08%

### The limit of SNR for the algorithm

We added noises with different level of signal-to-noise ratio (SNR) to give the limit bound of the algorithm. Experiments were also carried out both on authentic and synthesized data.

**Fig. 10:**
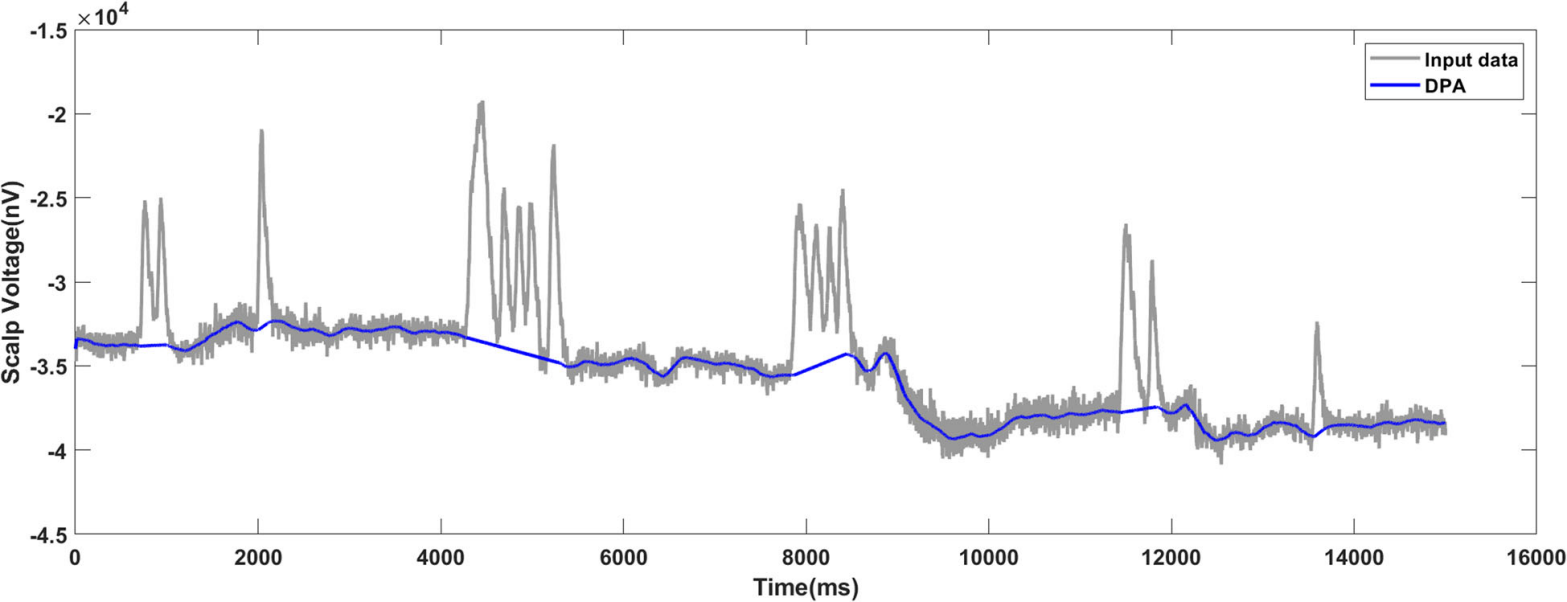
The DPA method applies on the raw data of motor imagery EEG and generates an accurate baseline

**Fig. 11:**
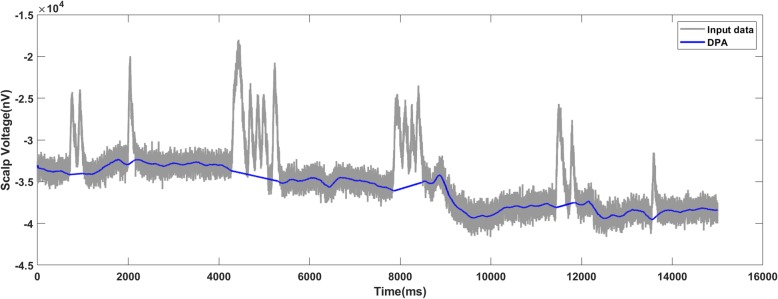
The DPA method generates a valid baseline on the data-trace with 7 dB SNR

**Fig. 12:**
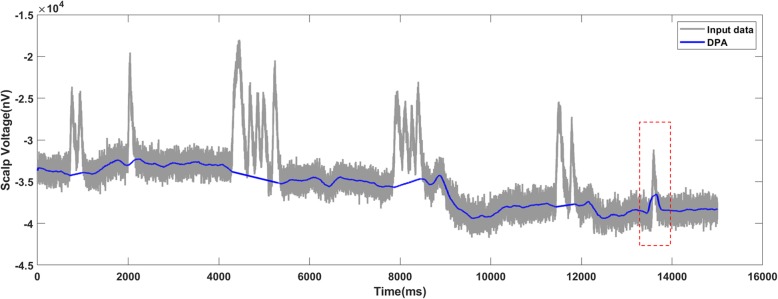
The DPA method applies on the motor imagery EEG data with 6 dB SNR. The detected baseline is with an inappropriate bump (marked by the red frame)

Figures 10, 11 and 12 present the results on motor imagery EEG data corresponding to the raw signal, added noise with SNR of 7 dB and with SNR of 6 dB respectively. From Fig. 11 where SNR is 7 dB for the added noise, the baseline detected by the DPA method was close to the one in the raw data. For the data with the SNR of 6 dB, there was a bump (marked by the red frame in Fig. 12) which implied some overcut. Therefore, the limit of the DPA in the data was set as 7 dB.

**Fig. 13:**
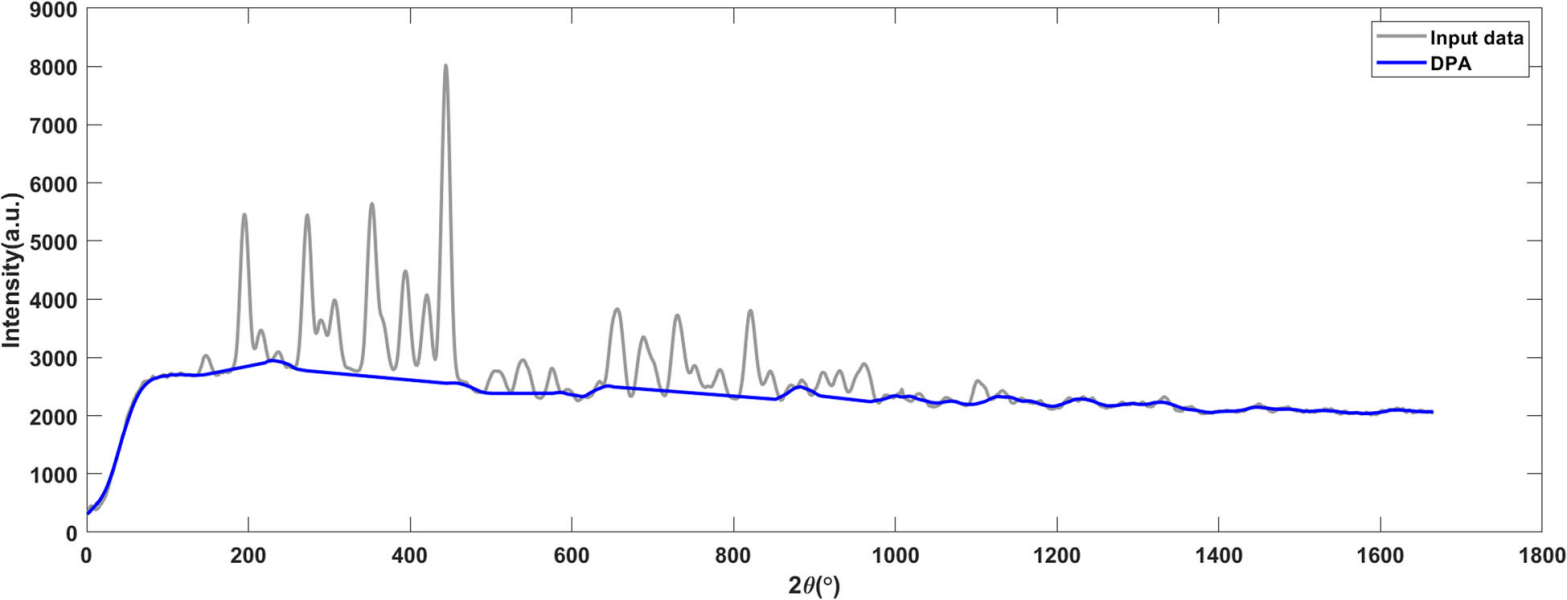
The DPA algorithm generates an accurate baseline on the raw X-ray diffraction data

**Fig. 14:**
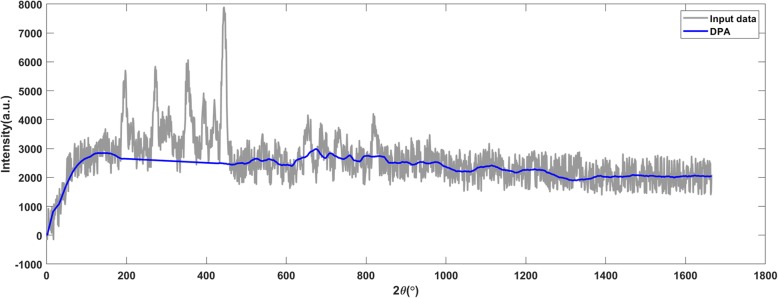
The DPA algorithm generates an accurate baseline on the raw X-ray diffraction data with added noises. The signal to noise ratio is 6 dB

**Fig. 15:**
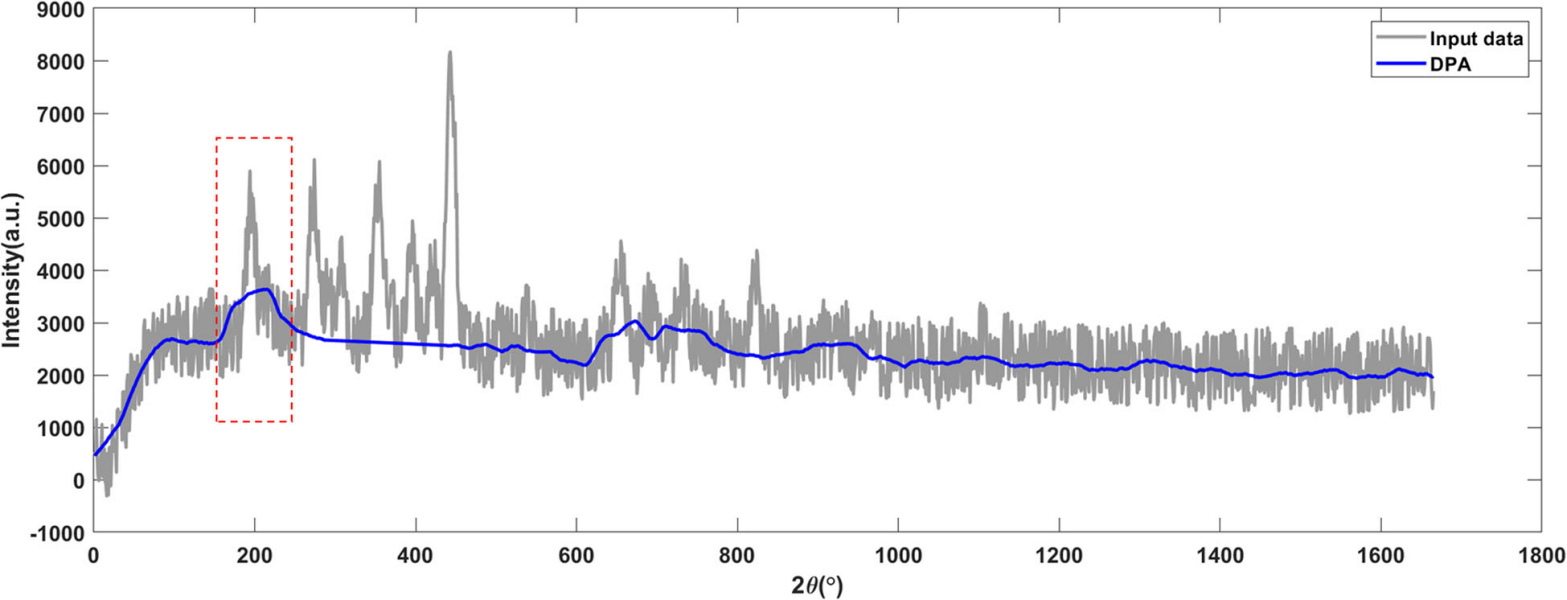
The DPA method applies on the X-ray diffraction data with 5 dB SNR. The detected baseline is with an inappropriate bump (marked by the red frame)

Figures 13, 14 and 15 present the results of X-ray diffraction data. The DPA algorithm generated accurate baseline on the raw X-ray diffraction data (shown in Fig. 13) and it was still valid when 6 dB noise was added (shown in Fig. 14). An overcut appeared when the SNR declined to 5 dB (shown in Fig. 15).

**Fig. 16:**
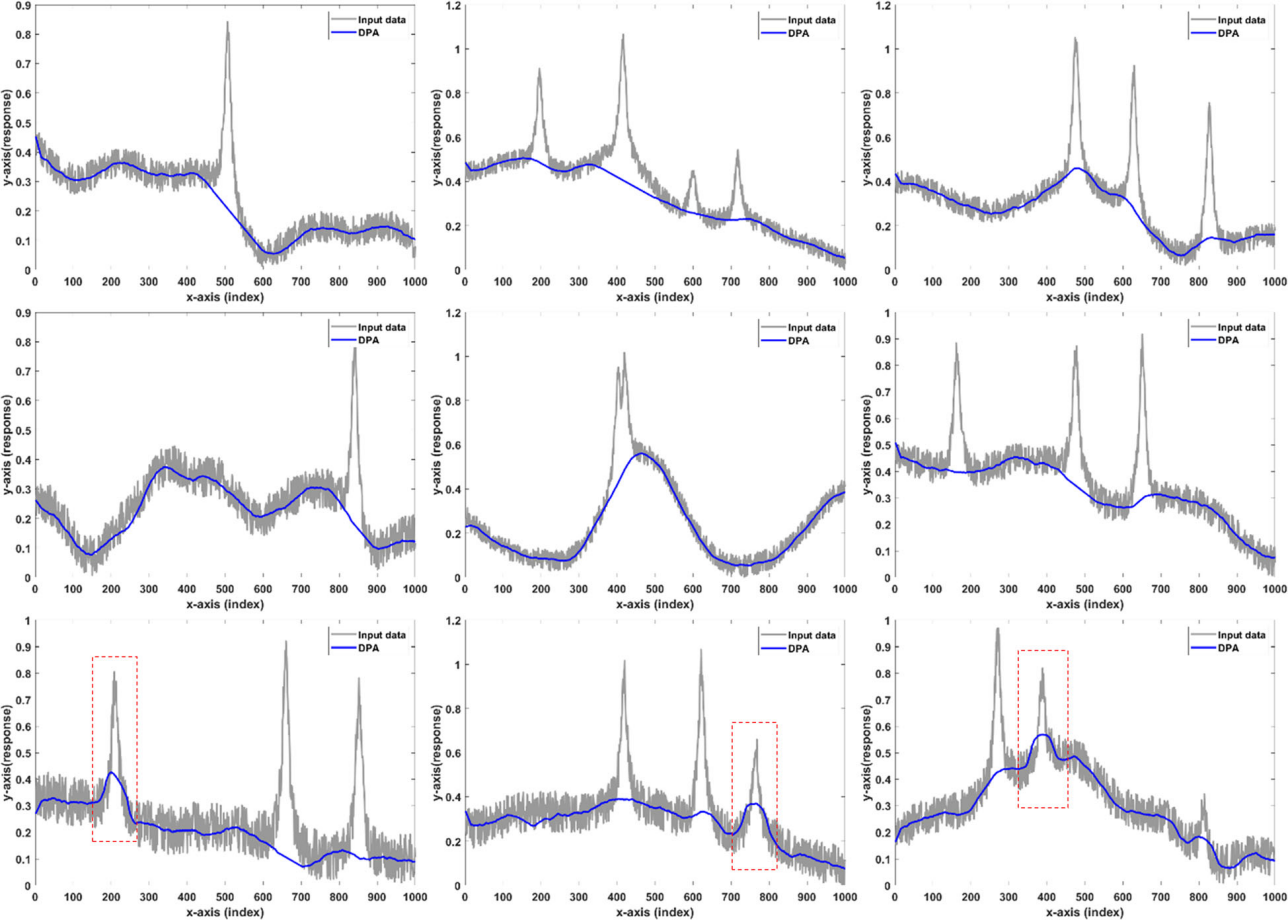
From 7 dB to 6 dB, DPA algorithm outputs reasonable baselines. When implemented on 5 dB data, DPA fails to produce accurate results due to the overcut marked in red frame. The first row: the baselines detected on the data with 7 dB SNR. The second row: results on the data with 6 dB SNR. The third row: results on the data with 5 dB SNR

Figure 16 presents the results generated on synthesized data with varying SNR. Subfigures from the first to the third row show the baselines detected on the data with 7 dB, 6 dB, and 5 dB SNR. The results showed that from 7 dB to 6 dB, DPA algorithm output reasonable baselines. When implemented on 5 dB data, DPA failed to produce accurate results due to the overcut marked in the red frame.

From these random testing results, we conclude that the DPA algorithm could perform well if the level of SNR was better than 7 dB. Since this value is an acceptable limit, we claim that the newly developed method is practical.

## Discussion

The testing results on both authentic and synthesized data indicated that the newly developed Derivative Passing Accumulation (DPA) method in this article outperformed other classical baseline detection methods. For the signals with random peaks oriented to the same direction, airPLS and DPA methods output similar results which were better than the others. For the data where signal peaks oriented towards both positive and negative directions (for example, the ECG signals), airPLS failed while EMD, wavelet and DPA methods performed well. Generally speaking, the DPA method was wider applicable and more stable. It generated accurate baselines in most cases. We have also tested the limitations of the new method. Noises in different ratios were added to the raw signals and it was found that DPA worked well under at least 7 dB, which was a practical level.

The DPA method was not able to produce valid results when the signal peaks were not fully recovered in the accumulated derivative form. In this case, the position belonging to the signal interval might be identified as the background point. That’s why, when the DPA algorithm failed, the result was always overcut. Sometimes the accumulated form was sufficient. However, the slight misplacement of the identified signal interval led to an obvious inappropriate bump in the generated baseline. The uncertainty may be a main drawback of the DPA method.

## Conclusions

Signal processing plays an important role in biological data analysis. It has a strong impact on the accuracy of downstream operations leading up to the analysis output. The new method developed in this article was able to simultaneously implement baseline removal and peak detection, which constituted the main content of the signal processing stage. Relying on the simple passing accumulation procedure and with the aid of the non-maximum suppression strategy, the proposed DPA method could conduct rapid and automatic calculations.

The results of comparison with the different algorithms that were applied to real-life biological data showed that the new method was more robust in a wide range of applications. We further measured the processing performance by testing the peak area loss rate for the synthesized data. The results also indicated that the DPA method had a superior accuracy. In addition, the operation under the passing accumulation also revealed its potential value for processing higher dimensional scenarios beyond the data stream. Further applications in image processing and the comprehension of higher dimensional mathematical meanings could be studied in future.

## Methods

**Fig. 17:**
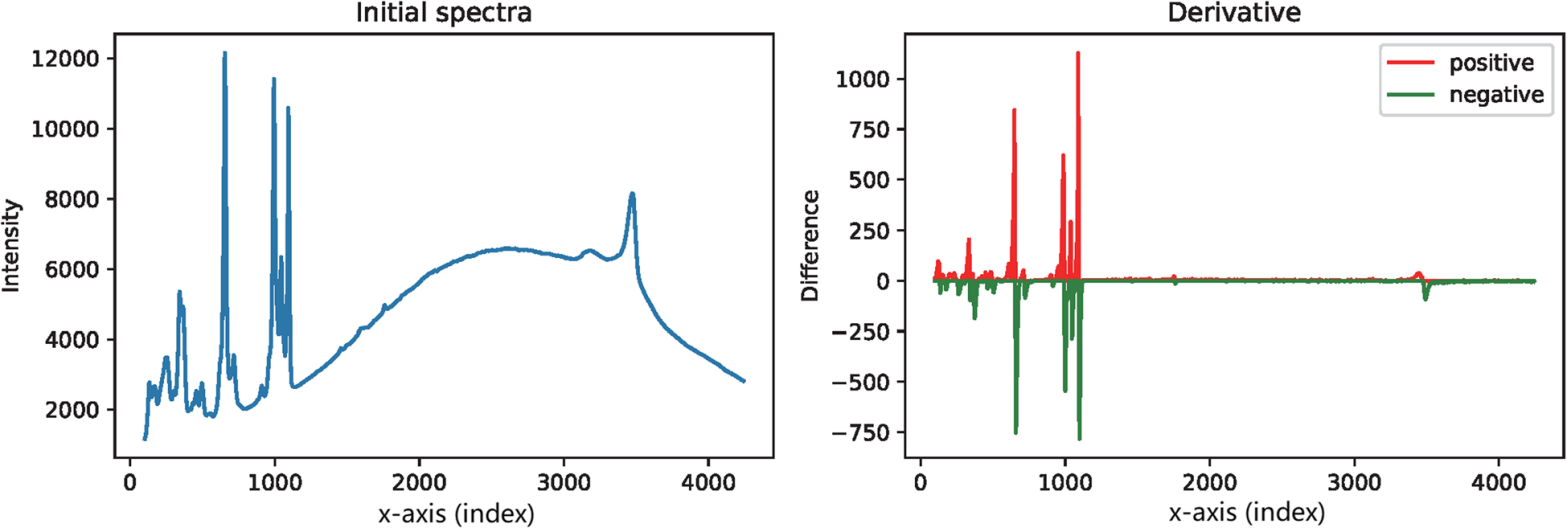
The plot of the derivative. Left: the initial raw spectra data (The x-axis can be assigned with any independent variable, or simply use the index of the point in the data sequence). Right: the plot of the derivative on each data point. The derivatives are categorized into positive part and negative part

The derivative is a local variable quantity that is not influenced by baseline drift. Explicitly, the background fluctuation is very small in a narrow interval, which could be omitted when considering the derivative. Only the local rise and fall apparently affect the derivative, so it is mainly dominated by the signal peaks. Here, we adopted the easiest operation of simple differences to obtain the discrete derivatives of the waveform. When we applied the differential operator on the data-trace, the rising interval had a positive ascent and the falling interval had a negative descent. The crest was the watershed that laid between the rising and falling parts. A practical data-trace illustrating this basic fact is presented in Fig. 17.

The first-order derivative trace was divided into positive and negative parts, which are denoted as P and N, respectively. Both parts carried the information of the initial data-trace reflecting the signal shape and location, but the baseline drift was eliminated. The basic idea of the new algorithm was utilizing this derivative to reconstruct the signal peaks and estimate their locations to discriminate the signal and background intervals. The procedure was explained as follows and formally described in Algorithm-1.

**Fig. 18:**
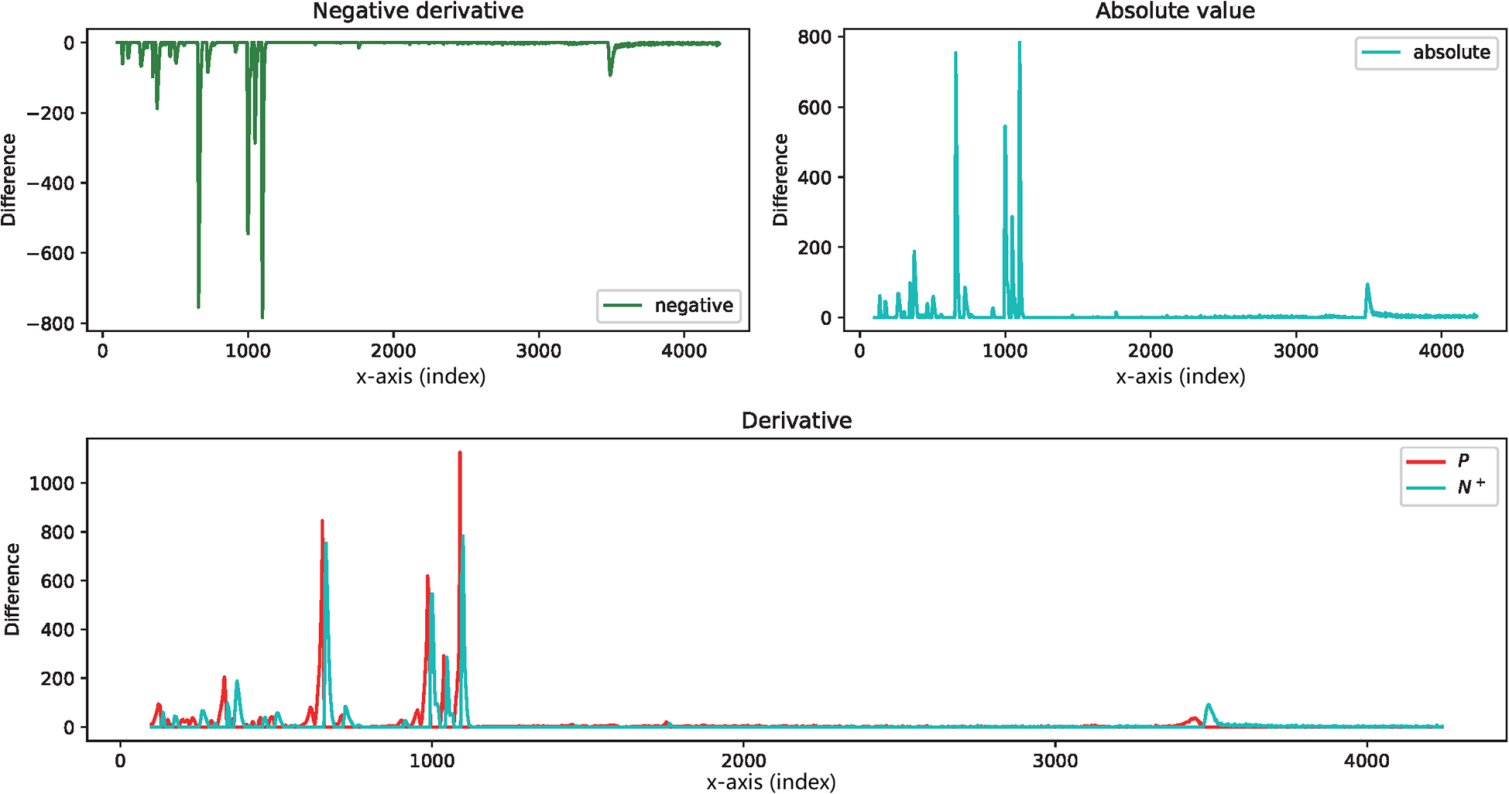
Upper left: the negative part N, upper right: flip N to get its positive version of N^+^, and bottom: overlay the trace of P and N^+^ at the same position

For the negative part N, its absolute value was used to flip the trace to be positive, as shown in Fig. 18 (the green line in the upper left of Fig. 18 is the negative part that is denoted as N, and the cyan line in the upper right of Fig. 18 is the flipped absolute curve). The flipped part is denoted as N^+^.

**Fig. 19:**
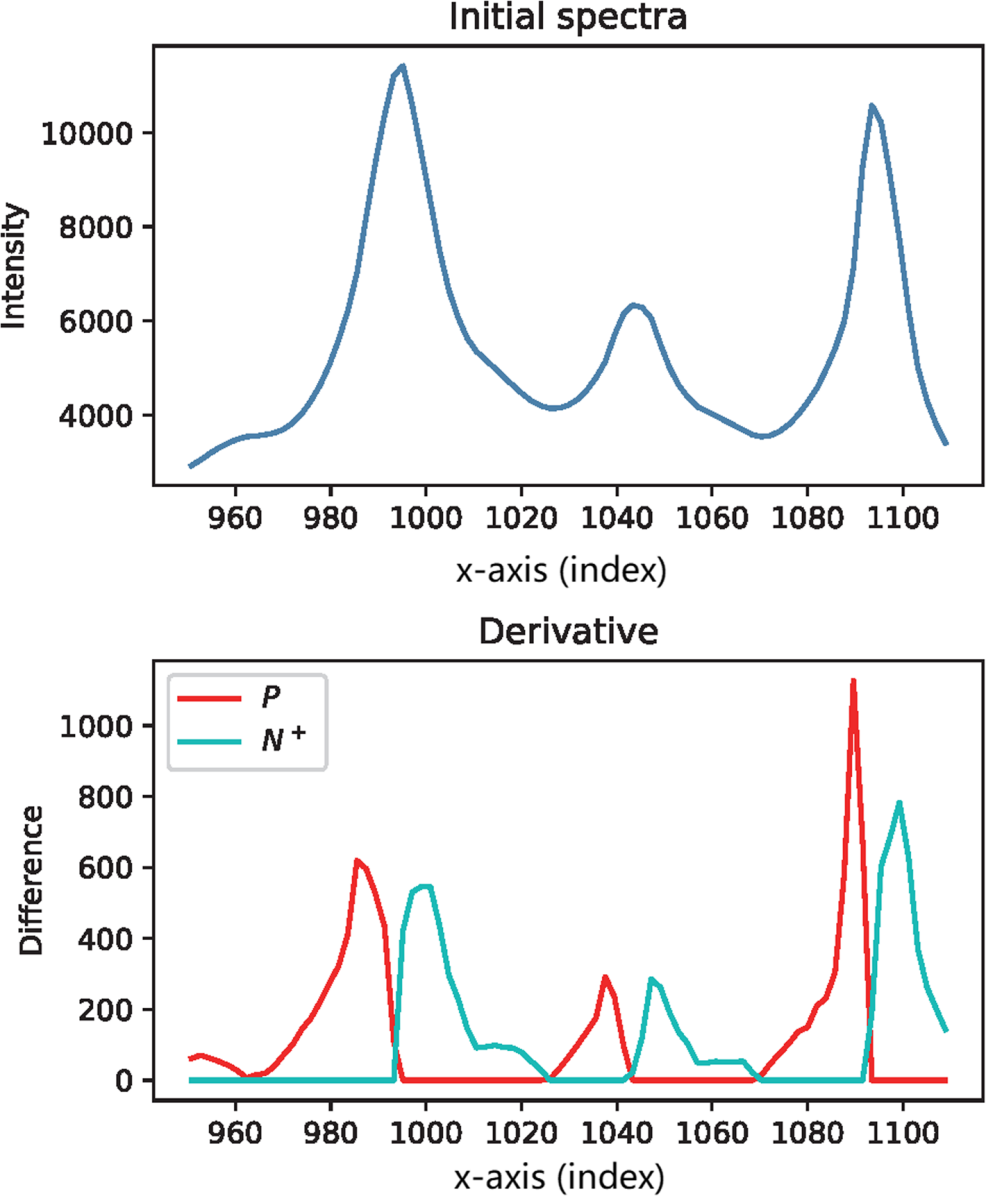
Compare the initial trace with the overlaid derivatives. Upper: an arbitrary trace representing the general raw signal data. The x-axis could be assigned with any independent variable, or simply use the index of the point in the data sequence. Bottom: the overlay of the positive part *P* and the negative part *N*^+^ of the derivative. Remark: the overlaid diagram forms a profile similar to the initial trace. The fact inspired the idea of using the two parts of the derivative to reconstruct the signals

While overlaying the two vectors P and N^+^, it is easy to see that the derivative traces look like steady peaks that are split from the initial trace at the crests (as shown in Fig. 19). Moreover, the interval of these peaks falls in the range of the corresponding initial peaks.

### Derivative Passing Accumulation

The key operation for rebuilding the signals is to shift and accumulate the trace of P and N^+^.

**Fig. 20:**
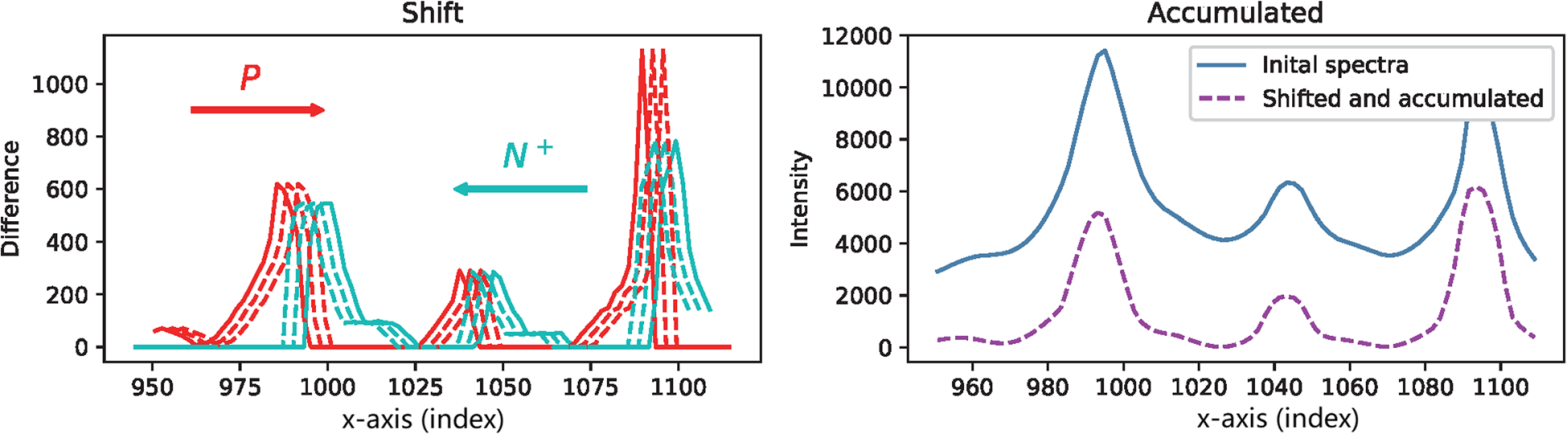
The passing accumulation operation. Left: slide positive part and negative parts toward each other to pass through. The value is accumulated on each shift. Right: the accumulated result compared with the initial raw spectra

**Fig. 21:**
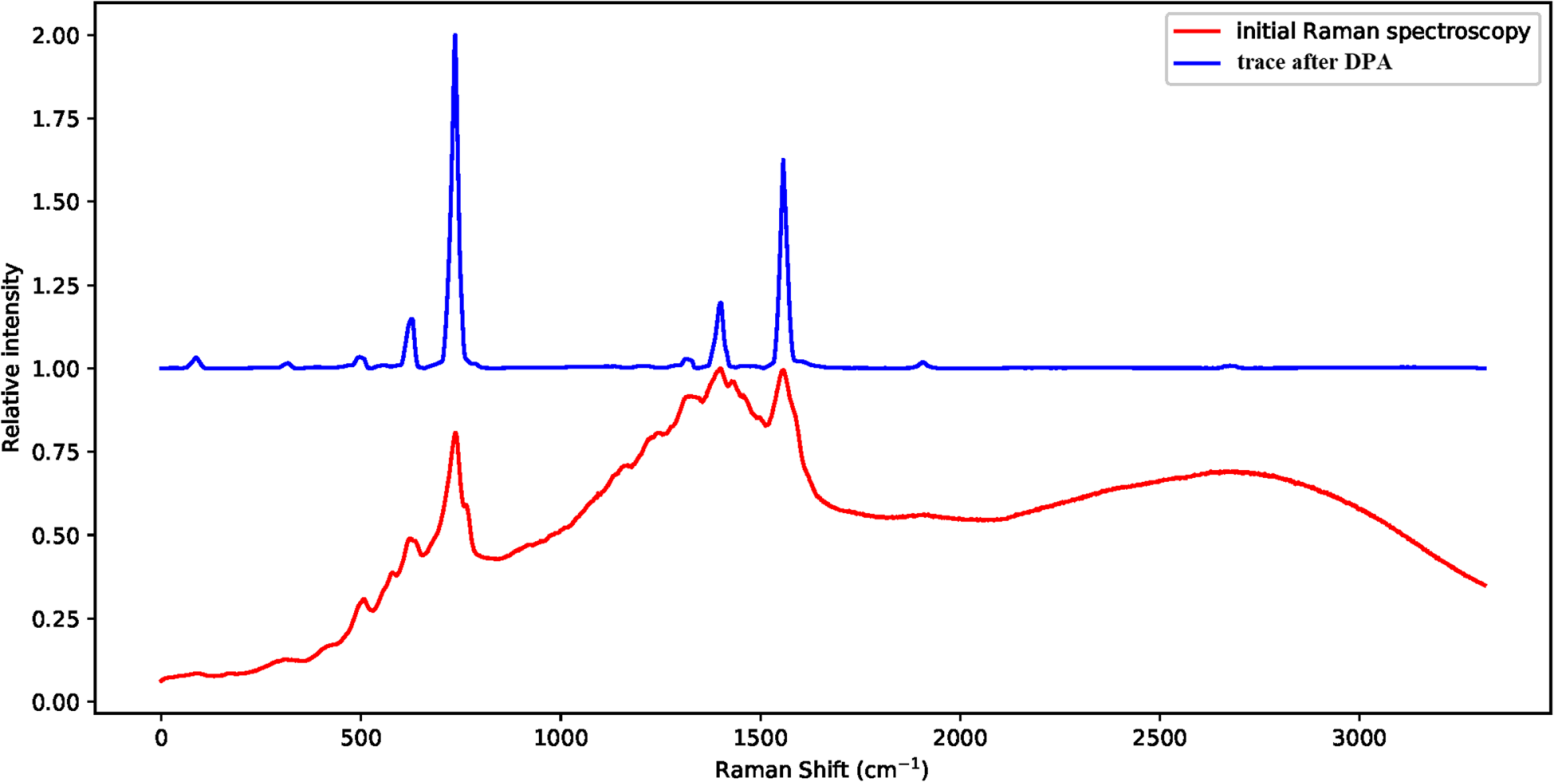
The effect of DPA computation applied on an authentic Raman spectroscopy. Compared with the initial trace, the peaks are kept and augmented at the same position and the baseline is straightened

The procedure is named the passing accumulation operation and the schematic diagram is presented in Fig. 20. The detailed calculation is given as follows. A shift width *k* is designated. Set α = {a_0_, a_1_, · · ·, a_m_}, and initialize a_i_, where *i* = 0, ⋯, *m*, to 0. Move *P* = {*p*_*0*_*, p*_*1*_*, · · ·, p*_*m*_} and N^+^ = {n_0_, n_1_, · · ·, n_m_} to each other *w* + 1 times and accumulate the result as α. In each step j, *a*_*i*_ *= a*_*i*_ *+ p*_*i − j*_ *+ n*_*i + j*_. Thus, the result of our passing accumulation is presented by Eq. (1)

1 $$ {a}_i=\sum \limits_{j=0}^w{p}_{i-j}+\sum \limits_{j=0}^w{n}_{i+j} $$

where *w* is the maximum shifting width and is manually set up. The newly defined computation is named as the derivative passing accumulation and denoted as DPA for short. The effectiveness of this computation is presented in Fig. 21, in which it could be intuitively seen that the data-trace of a Raman spectroscopy with serious baseline drift is straightened and the signal peaks are kept and augmented. The accumulating procedure is summarized in Algorithm-1.

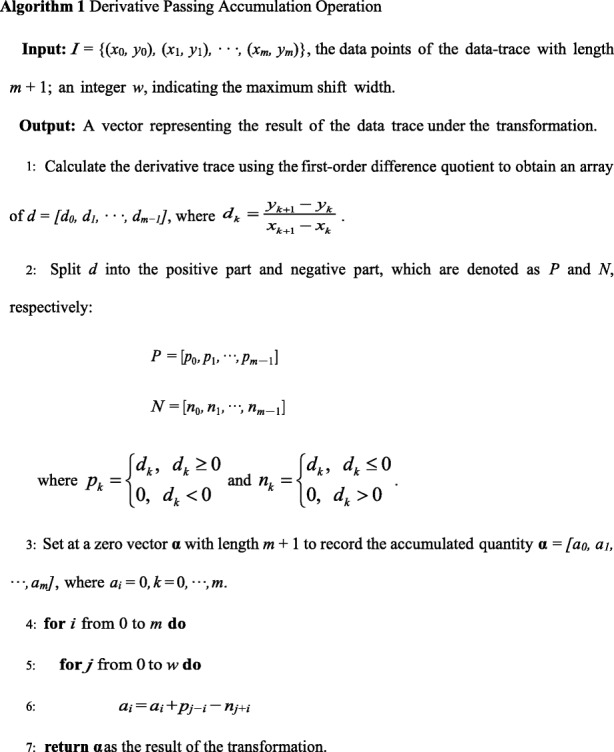



### Automating

In the preliminary version of the algorithm, the maximum shift width *w* was set manually and uniformly. In the following upgraded version, the non-maximum suppression was applied to automatically determine *w* for each subscript in the derivative array, which thereby improved the whole algorithm to become fully self-driven. Non-maximum suppression (NMS) is a widely used technique in computer vision tasks, such as the Faster R-CNN [24]. The basic idea is to select the object according to its descriptive value, i.e. to examine if its value was the maximum among all those intersecting with it. In our case, this principle could be explained as follows.

On a fixed point (*x*_*i*_, *y*_*i*_) in the data-trace, for each width *w*, we could compute the accumulation according to Eq. (1) to get a function with respect to *w*,

2 $$ A(w)=\sum \limits_{j=0}^w{p}_{i-j}+\sum \limits_{j=0}^w{n}_{i+j} $$

We would like to determine the width *w*_*i*_ automatically for position *i* (corresponding to the data point (*x*_*i*_, *y*_*i*_)). According to the principle of non-maximum suppression, we select *w*_*i*_ which meets the requirement of Eq. (3),

3 $$ A\left({w}_i\right)=\underset{\mid t-i\mid \le {w}_i}{\max }A\left({w}_t\right) $$

Substituting (2) into (3), we get that the width *w*_*i*_ must satisfy the condition in (4),

4 $$ {w}_i=\left\{k|k\in {Z}^{+},\sum \limits_{j=0}^k{p}_{i-j}+\sum \limits_{j=0}^k{n}_{i+j}=\underset{\left|t-i\right|\le k}{\max}\left(\sum \limits_{j=0}^{w_t}{p}_{t-j}+\sum \limits_{j=0}^{w_t}{n}_{t+j}\right)\right\} $$

We just need to determine an appropriate *w*_*i*_ according to Eq. (4) instead of finding out all the possible solutions. To implement the procedure in the program, we can execute the accumulation adaptively by checking if the requirement is met during the passing. In the computation according to Eq. (1), the summation for each index *i* could be implemented as follows. Set *a*_*i*_ = 0 and start a loop to grow *j* from 0 to $\frac{L}{2}$ (*L* represents the length of the derivative array). Accumulate the *p*_*i* − *j*_ and *n*_*i* + *j*_ to *a*_*i*_. For each step in the loop, examine if the accumulated value was maximum in its *j*-nearest neighborhood. When the maximum requirement is not satisfied, the accumulation at this index stops and the accumulated value is stored. It also stops if all the *j* nearest neighbors stop growing. The corresponding width *w* is set equal to *j* consequently. The adaptive passing accumulation is accomplished as the termination of the loop or every width *w* is determined.

We summarized the scheme in Algorithm-2.

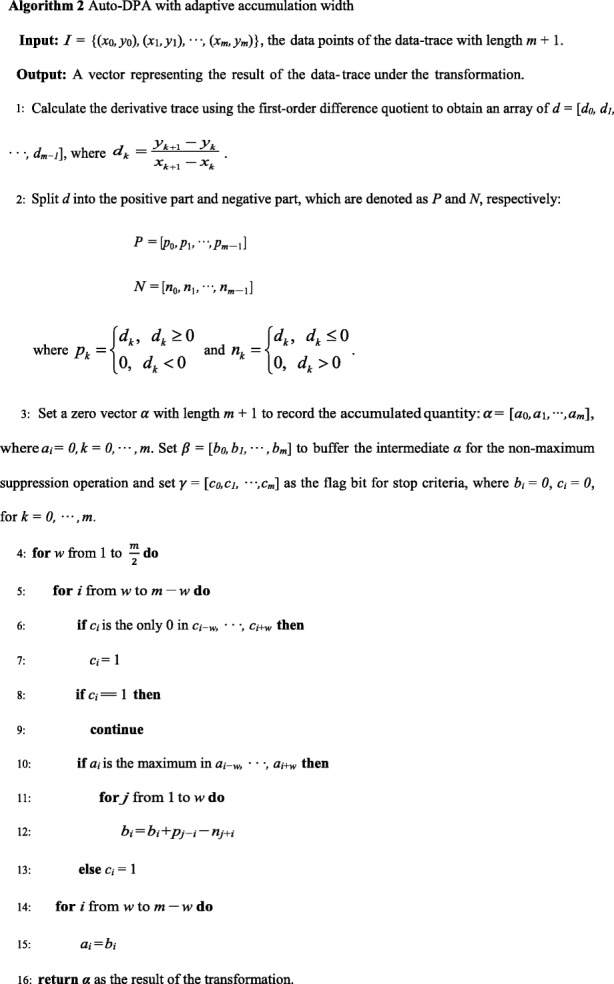



### The complete algorithm

**Fig. 22:**
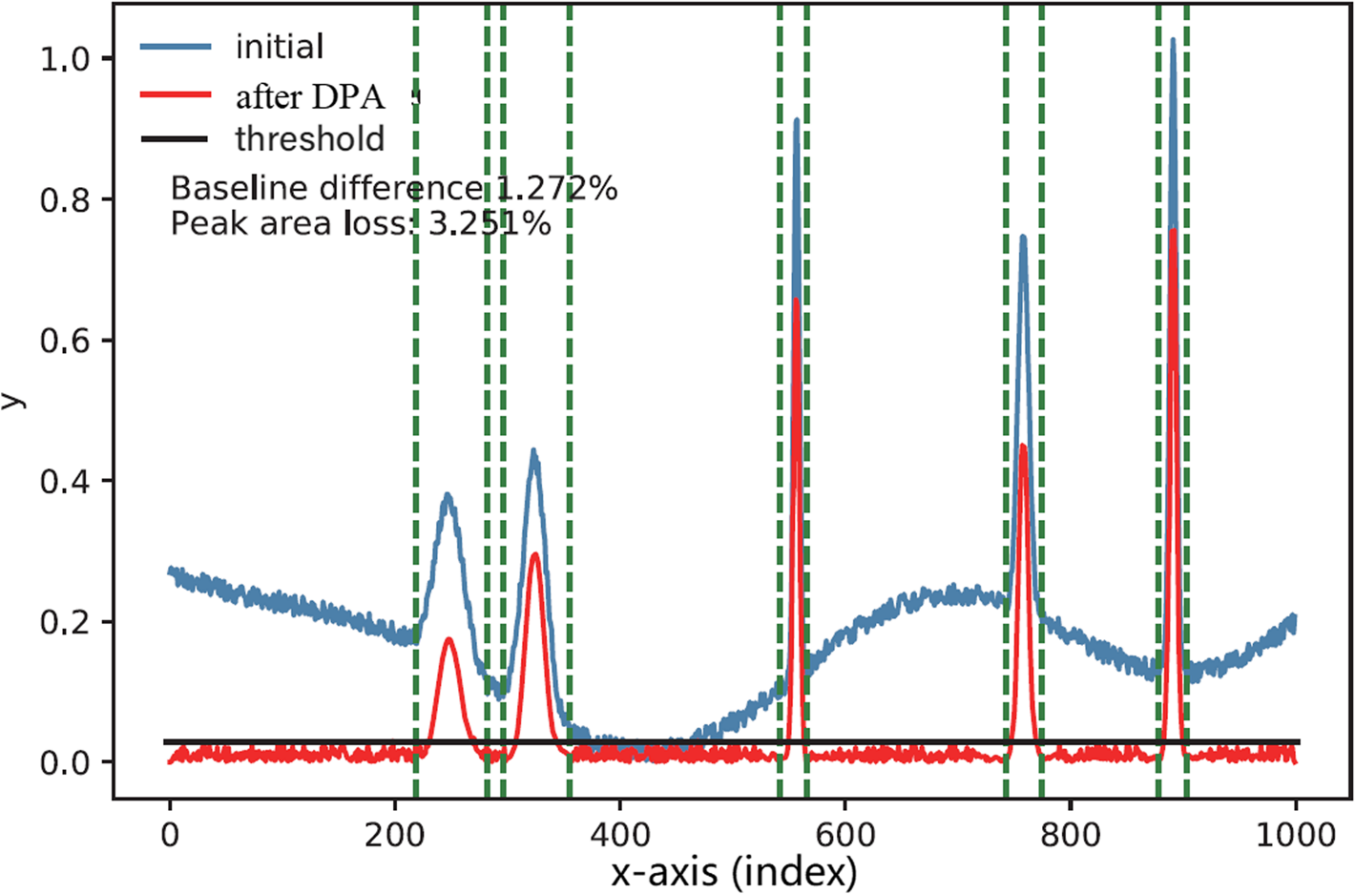
The peak identification results. Set a threshold on the result after DPA operation, in which the baseline is removed and signals are kept. Then, the positions of peaks are identified and the points belonging to the baseline in the initial trace are extracted

With the help of non-maximum suppression strategy, the DPA was upgraded into an automatic pipeline. With the converted waveform *T*, it was straightforward to get the final results. In *T*, since the baseline drift was removed and the peaks were kept in the corresponding interval, we just needed to select a threshold [25] to divide the array into peak points and baseline points. In this way, the signal peaks were extracted. In addition, the baseline could be constructed by linearly connecting the key points that were selected according to the identified baseline points. Figure 22 illustrated the schematic diagram.

The complete procedure was summarized in Algorithm-3 and named as the derivative passing accumulation method (DPA).

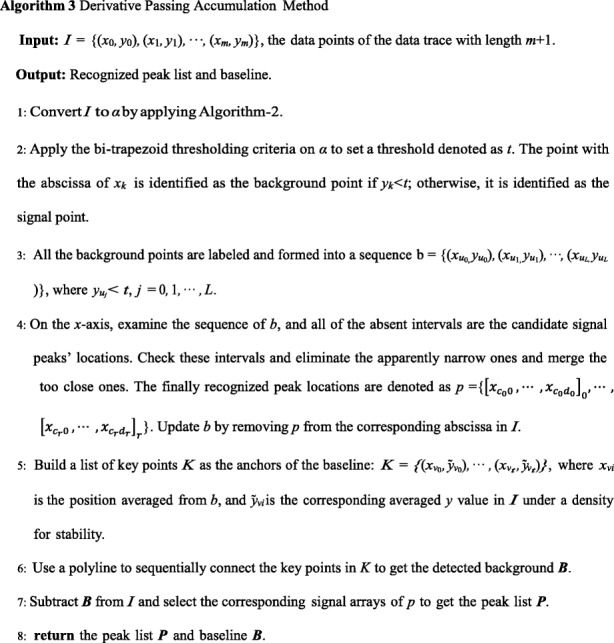



## Perspectives

In future, studies will be carried out in two directions.

First, we plan to improve the performance based on fractional derivative techniques. Because, currently there is a trend of exploiting fractional derivatives for solving different identification problems. These show better results than standard first-order derivative-based algorithms [26–28]. If we find a way to utilize the fractional derivative for recovering the signals, a more accurate separation of the baseline intervals will be achieved, leading to better results.

Second, we will work on extending the application of the DPA method to a 2-dimensional case. We will study to discover the way of appropriately defining the accumulation of derivatives for 2-D function. Thus, develop the scheme for extracting the background, based on the accumulated results. Consequently, the upgrade to a higher dimension will enable the DPA strategy to process image data and expand the application range.

## Supplementary information

**Additional file 1.** Testing data of the mass spectroscopy, infrared spectroscopy and energy curve of audio.

## Data Availability

The datasets used to generate the figures presenting results on Raman spectra, ECG profiles were downloaded from public databases which were cited within the manuscript. The data used to generate the figures presenting results on the energy curve of the audio, mass spectroscopy and infrared spectroscopy are available in the Additional file 1. The synthesized data for random testing were generated following the procedure explained within the manuscript.

## Abbreviations

DPA: Derivative Passing Accumulation method
ECG: Electrocardiograms
EEG: Electroencephalograms
